# Editorial: Antimicrobial resistance and one health: from culture to genomics

**DOI:** 10.3389/fcimb.2023.1294241

**Published:** 2023-10-10

**Authors:** Akebe Luther King Abia, Asfatou Ndama Traore, Natasha Potgieter

**Affiliations:** ^1^ Antimicrobial Research Unit, College of Health Sciences, University of Kwazulu-Natal, Durban, South Africa; ^2^ Environmetal Research Foundation, Westville, South Africa; ^3^ Department of Biochemistry and Microbiology, Faculty of Science, Engineering and Agriculture, University of Venda, Thohoyandou, Limpopo, South Africa

**Keywords:** antibiotics, antimicrobial resistance, genomics, one health, pathogens, public health, circular health

Antimicrobial resistance (AMR) is among the leading global public health threats of the 21st century. Thus, AMR threatens human, animal, and environmental health globally (Abia and Essack, 2023). Moreover, the widespread misuse and uncontrolled prescription of antibiotics aggravates the problem, diminishing antibiotic efficacy, including last-resort ones (Lu et al., 2023). This problem is accentuated in rural settings in low- and middle-income countries where community members are small-scale farmers, often living with their livestock inside the yards and lacking adequate water and sanitation facilities (Sulis et al., 2022). Inadequate animal and human waste disposal from household and agricultural practices could transfer antimicrobial-resistant pathogens and their associated genes to the environment. These pollutants may pose a health risk to resource-limited community members upon exposure to such polluted environments. Therefore, monitoring antimicrobial resistance requires a one health approach involving the human, animal, and environmental sectors. Furthermore, employing advanced genomic tools to complement existing culture-based techniques would provide a more comprehensive appreciation of the extent of AMR globally. Therefore, the current Research Topic aimed at providing an update on the one health approach to AMR using cultural and genomic approaches. This Research Topic includes seven articles by 45 authors from eight countries in the Americas, Asia, and Europe.

The development of resistance in bacteria is a complex process involving several mechanisms. Ramamurthy et al. reviewed the genetic network and programmed regulations used by bacterial pathogens in antimicrobial resistance. Mechanisms identified could be cellular (outer membrane modifications, enzyme inactivation, biofilm formation, and efflux pump activation) and/or molecular (gene mutations and upregulation, quorum-sensing networks, and other mechanisms). Once developed, mobile genetic elements (e.g., plasmids, integrons, and insertion sequences) mostly aid in disseminating AMR in the environment, influenced by the presence of stressors such as biocides, antibiotics, heavy metals, and detergents.

Children are vulnerable to infections due to their less developed immune systems. Patil et al. assessed the resistance genomics and molecular epidemiology of *Pseudomonas aeruginosa* in young children, specifically targeting high-risk extended-spectrum beta-lactamase (ESBL)-producing clones. The authors analysed 294 clinical isolates from a paediatric hospital in China using culture-dependent and independent methods and found that 56%, 40%, 39%, 36%, 33%, and 32% of the isolates were resistant to piperacillin-tazobactam, cefepime, ceftazidime, imipenem, meropenem, and ciprofloxacin, respectively. Furthermore, 42% of the isolates were ESBL producers. Polymerase chain reaction and sequencing revealed the presence of numerous genes including *bla*_CTX-M-15_, *bla*_NDM-1_, *aac*(3)IIIa, *tet*(A), and 23 sequence types, as well as a *de novo* strain. The isolates also harboured various plasmids that could aid in the transmission of these resistance genes. Still in humans, Jia et al. genomically characterised bacteraemia-associated carbapenem-resistant *Serratia marcescens* isolates and reported the emergence of KPC-2-encoding IncR plasmids in a clinical setting to understand their resistance and transmission dynamics. After sequencing the complete genome of two *S. marcescens* isolates, the authors observed that the isolates harboured *bla*_KPC-2_-bearing IncR plasmids and multiple plasmid-borne antimicrobial resistance genes. Comparative plasmid analysis further suggested common ancestral lineage for both isolates and concluded that the plasmid borne by both isolates could hinder the transmission of KPC-2-producing *S. marcescens* in clinical settings.

While some clinical bacteria may acquire resistance traits due to exposure to external stressors, others are intrinsically resistant to several antimicrobials. Hence, Shi et al. functionally characterised a novel aminoglycoside phosphotransferase, APH(9)-Ic, and its variant from *Stenotrophomonas maltophilia*. After performing recombinant protein expression and enzyme kinetic studies, the authors used whole-genome sequencing to determine the genetic context of the studied gene. The authors observed that *aph*(9)-Ic and *aph*(9)-Ic1 conferred spectinomycin resistance. Furthermore, a recombinant strain harbouring *aph*(9)-Ic displayed a marked increased minimum inhibitory concentration level against spectinomycin compared with the control strains; *aph*(9)-Ic was chromosome-borne, with a relatively conserved genetic environment and no mobile genetic element in its surrounding region.

The one health approach has been shown to provide a comprehensive understanding of the spread of AMR in the human–animal–environment triad. Thus, Yasmeen et al. investigated the occurrence of ESBL-harbouring *Klebsiella pneumoniae* from 793 samples collected from animals, humans, and the environment. Their study found K. pneumoniae in 11.6%, 8.4% and 7.0%, of animal, humans, and environmental samples, respectively, with the animal isolates harbouring more ESBL genes (mostly *bla*_SHV_) than their human and environmental counterparts. These results show the potential spread of these pathogens within the three one health compartments.

Given the debating effects of AMR, involving humans, animals, and the environment, attempts at curbing this ill must take a holistic approach involving multisectoral stakeholders. Thus, Ahmad et al. reviewed how the one health concept has been implanted and evolved in combating AMR. They identified that pillars of support to combat AMR include instituting robust ABR surveillance in the various sectors individually and in combination and at national and international level, as well as improving laboratory resources and executing and strictly implementing core plans and actions. Furthermore, drawing on lessons from the recent COVID-19 pandemic, Mantegazza et al. proposed a *de novo* approach based on a convergence model to fight AMR, exploiting the current Sustainable Development Goals (SDG) roadmap, emphasising a circular approach and highlighting win–win scenarios by enforcing existing objectives and targets to strengthen a multidisciplinary synergistic effort towards fighting AMR. They argued that the global importance of AMR warrants that it be an integral part of the SDG roadmap, involving all the SDG objectives, as such efforts could significantly advance health as a system by focusing on a major priority such as AMR.

Collectively, these studies contribute to knowledge on the spread of AMR in humans, animals, and the environment. Furthermore, they reveal the various mechanisms involved in AMR in bacteria, including factors triggering their occurrence and factors favouring such spread. Therefore, combining culture and advanced molecular techniques such as genomics would paint a brighter picture regarding the overall AMR burden. Put together, the studies provide evidence that adopting a one health concept for a successful fight against AMR is imperative.

## Author contributions

AA: Conceptualization, Methodology, Writing – original draft, Writing – review & editing. AT: Conceptualization, Methodology, Writing – review & editing. NP: Conceptualization, Methodology, Writing – review & editing.
